# Production of *Escovopsis weberi* (Ascomycota: Hypocreales) Mycelial Pellets and Their Effects on Leaf-Cutting Ant Fungal Gardens

**DOI:** 10.3390/pathogens12020330

**Published:** 2023-02-15

**Authors:** Thais Berçot Pontes Teodoro, Aline Teixeira Carolino, Raymyson Rhuryo de Sousa Queiroz, Patrícia Batista de Oliveira, Denise Dolores Oliveira Moreira, Gerson Adriano Silva, Richard Ian Samuels

**Affiliations:** Laboratório de Entomologia e Fitopatologia, Centro de Ciências e Tecnologias Agropecuárias, Universidade Estadual do Norte Fluminense Darcy Ribeiro, Campos dos Goytacazes 28013-602, RJ, Brazil

**Keywords:** liquid culture, mycoparasite, pest control, baits, formicidae, biological control

## Abstract

The maintenance of the symbiosis between leaf-cutting ants and their mutualistic fungus *Leucoagaricus gongylophorus* Singer (Moller) is vital for the survival of both species. The specialist fungal parasite *Escovopsis weberi* Muchovej & Della Lucia is a threat to this symbiosis, causing severe damage to the fungal garden. Mycelial pellets are resistant fungal structures that can be produced under laboratory conditions. These structures were studied for use in biological pest control, but the production of mycelial pellets has not previously been documented in *Escovopsis*. One of the aims of this study was to induce *Escovopsis weberi* to produce mycelial pellets and investigate the potential of these pellets for the control of leaf-cutting ants. We compared the pathogenicity of *Escovopsis weberi* mycelial pellets and conidia against mini-colonies of *Acromyrmex subterraneus subterraneus* Forel when applied in the form of baits. Worker ants were able to distinguish mycelial pellets from conidia, as baits with mycelial pellets were more attractive to workers than those with conidia, causing a greater negative impact on colony health. All types of baits containing *Escovopsis weberi* influenced the foraging activity but only treatments with viable fungal propagules resulted in an increase in the quantity of waste material, with a significant negative impact on the fungal garden biomass. The results provided novel information regarding *Escovopsis* recognition by worker ants and differences between conidia and mycelial pellet dynamics in leaf-cutting ant colonies, with new perspectives for the biological control of these important pests.

## 1. Introduction

Leaf-cutting ants (Attini: Formicidae) are amongst the most important agricultural and forestry pests throughout the Neotropics [1,2]. They cause severe damage due to the large amount of fresh leaves they cut and carry to their nests, which they use to cultivate the symbiotic fungus *Leucoagaricus gongylophorus* (Möller) Singer (Agaricales: Basidiomycota) [3,4,5]. In this symbiosis, the ants provide the fungus shelter, fresh leaves, and protection against parasites; in exchange, *L. gongylophorus* provides the ants with easily assimilable nutrients via specialized hyphal structures called gongylidia, which are the main food resource for the queens, brood, and workers [6,7]. Additionally, gongylidia provide the ants with enzymes that are useful for degrading plant material [7,8].

Brazil is one of the most important wood producers in the world. Wood production represents the fourth largest crop when considered by area, consisting of mainly fast-growing monocultures, such as eucalyptus, pine, acacia, and teak [9,10]. However, leaf-cutting ants can cause serious damage to these plantations, especially at the sapling stage [11]. The defoliation caused by ants significantly reduces wood production, causing a decrease in tree growth and also making the trees more vulnerable to attack by other pests [12,13].

Despite being ecologically important in natural environments, where leaf-cutting ants improve soil penetrability, nutrient cycling, and seed dispersal [14,15,16,17], these insects are a major concern in agricultural and forestry systems. The genus *Acromyrmex* includes 35 species; however, only 6 species are important as pests, i.e., *A. balzani*, *A. octospinosus*, *A. rugosus*, *A. subterraneus brunneus*, *A. subterraneus molestans*, *and A. subterraneus subterraneus* [18,19]. *Acromyrmex subterraneus subterraneus*, which was studied here, is mainly found in Brazil, Argentina, and Paraguay.

Synthetic chemical insecticide baits continue to be the most common control method used against these ants [20]. Currently, sulfluramid, which is a persistent organic pollutant, is the most efficient insecticide for controlling leaf-cutting ants and it is widely used in Brazil [13], even though the Stockholm Convention banned its use. However, chemical control methods cause negative impacts on human health and the environment [21]. This insecticide was found to contaminate soil, eucalyptus leaves, and water (underground, river, and coastal waters), which is of great concern, mainly in relation to human exposure to this compound [22,23]. In addition, the use of chemical insecticides promotes pest resistance and negatively affects non-target organisms [24,25,26,27]. The search for alternative control methods that are not detrimental to the environment or human health will benefit agriculture, animal health, and the ecosystem. 

The use of biological agents could be an environmentally safe alternative for the control of leaf-cutting ants [2,28]. *Escovopsis weberi* (Ascomycota: Hypocreales) is a virulent specialist fungus that parasitizes *L. gongylophorus* and has the potential to be employed in biocontrol programs [29,30,31]. In this parasitism, *L. gongylophorus* is degraded by direct hyphal contact between the two fungi, aided by hook-like protuberances that consume nutrients from *L. gongylophorus* living cells, leading to host death and consequent colony collapse if the worker ants cannot control this parasite [31,32,33,34].

Despite the capacity of *Escovopsis* to rapidly over-run *L. gongylophorus* fungus gardens, the ants have developed strategies to prevent or deal with eventual infections. The ants possess metapleural glands that produce antimicrobial secretions [35,36], and they constantly weed and groom the gardens to remove any contaminants [37,38]. The presence of *Pseudonocardia*, which is a symbiotic bacterium, on the bodies of some species of worker ants produces antibiotics that reduce the chances of *Escovopsis* infections [30,39,40]. Even so, *E. weberi* is still frequently found attacking leaf-cutting ant colonies [41]. 

A diverse range of fungi can produce structures called sclerotia to survive challenging conditions [42]. Sclerotia are dense hyphal aggregations that are highly resistant to adverse conditions, such as the absence of the host or desiccation. These structures are viable for a longer period compared with conidia and can germinate whenever the environmental conditions become favorable [43]. In nature, sclerotia are commonly found, especially in the soil [44]. However, when cultured in liquid media under constant agitation, it is possible to induce certain fungi to produce structures similar to sclerotia. In many cases, these so-called microsclerotia [45,46,47] are probably better described as mycelial pellets (hereafter referred to as MPs).

Specialist mycoparasites, such as *Escovopsis*, have not previously been documented to produce such structures [48], and the application of *Escovopsis* MPs could be an interesting and novel approach to control leaf-cutting ants. Interestingly, *Escovopsis* mycelium, unlike conidia, are not susceptible to ants’ metapleural gland secretions, which are responsible for antimicrobial activity against a variety of pathogens [35]. MPs store large amounts of nutrients in their structures [43], which may promote faster development of the parasite when attacking leaf-cutting ant fungus gardens, and have an increased shelf life [44,49]. 

The aim of this study was to develop an alternative strategy for leaf-cutting ant control. The four main objectives were as follows: (1) to induce *E. weberi* to produce MPs; (2) to assess the reproductive capacity of MPs; (3) to assess the effect of *E. weberi* MP baits against *A. subterraneus subterraneus* mini-colonies when compared with baits containing *E. weberi* conidia; and (4) to study the acceptance of both propagules, viable or dead, when offered to the ants in the form of baits. The results showed the potential of *E. weberi* in the form of MPs for leaf-cutting ant control with specific advantages over the application of conidia. 

## 2. Materials and Methods

### 2.1. Field Collection of Colonies, Maintenance, and Mini-Colony Establishment

*Acromyrmex subterraneus subterraneus* colonies were collected in Bom Jardim, Rio de Janeiro State, Brazil (22°13′16.37″ S, 42°15′14.74″ W; 709 m a.s.l.). This region is a tropical montane forest, located near urban and agricultural areas. We collected whole colonies after gently excavating the soil around each colony. Fungus gardens were transferred to clean containers with as many ants as possible, including the queens. The colonies were maintained in the Myrmecology Unit at the Universidade Estadual do Norte Fluminense Darcy Ribeiro (UENF). *Acromyrmex* species identification followed the keys provided by Fowler et al. [50]. The colonies were offered fresh *Acalypha wilkesiana* leaves on a daily basis and kept at a controlled room temperature (26 °C) and 70% relative humidity. For the bioassays, queenless mini-colonies were established randomly from the collected colonies. Each mini-colony was composed of ants of all 4 castes in a 350 mL of fungus garden inside a clear plastic pot that was placed in a white plastic tray (40 cm × 30 cm × 10 cm) that was maintained in the dark at room temperature and 70% RH.

### 2.2. Escovopsis Isolation, Identification, and Conidial Production

*Escovopsis weberi* (AT-02; NCBI accession numbers: OQ345555 and OQ345556) was isolated from an *Atta sexdens rubropilosa* colony collected in a eucalyptus plantation in São Francisco do Itabapoana (21°26′57″ S, 41°11′51″ W; 50 m a.s.l.), Rio de Janeiro State, Brazil, and maintained in the Entomology and Phytopathology Department (UENF) Pathogenic Fungi collection. In order to establish a pure culture of this isolate, single spores were cultured as described by Choi et al. [51].

After single spore culturing, AT-02 was inoculated on potato dextrose agar (PDA) culture medium in Petri dishes (9 cm diameter) and maintained in the dark at 27 °C and 70% RH. After 5 days, spores were harvested with the aid of a spatula and suspended in Tween 80 (0.03% in sterile distilled water). Conidial concentrations were estimated using a Neubauer hemocytometer and a suspension of 1 × 10^7^ conidia mL^−1^ was used after serial dilution. This concentration was either used for MP production or for making baits containing conidia that were used in the subsequent bioassays.

### 2.3. Mycelial Pellet Production in Liquid Culture 

*Escovopsis weberi* AT-02 MP was grown in liquid culture following a protocol developed by Carolino et al. [52]. The medium consisted of chickpea flour (3%), yeast extract (4%), and dextrose (4%) in distilled water. The procedure was as follows: First, the chickpea flour and distilled water were autoclaved for 20 min at 121 °C and then filtered using Miracloth (Sigma-Aldrich, Barueri, São Paulo, Brazil). Then, yeast extract and dextrose were added to this infusion. The culture medium was then autoclaved again.

Forty-nine milliliters of sterile liquid culture medium were added to 250 mL sterile Erlenmeyer flasks with 1 mL of conidial suspension (1 × 10^7^ conidia mL^−1^). The flasks were placed in an orbital shaker (Solab®, Piracicaba, São Paulo, Brazil, model SL-223/F) in the dark at 27 °C and 230 rpm for 4 days. At 24 h, 48 h, 72 h, and 96 h post-inoculation, 1 mL aliquots were taken from the culture medium to evaluate MP development and morphology. After 4 days, MPs were washed 5 times with sterile distilled water and centrifuged at 3000 rpm for 5 min (Nova Técnica®, Piracicaba, São Paulo, Brazil, model NT 810). Fresh MPs were stored at 4 °C. To determine the MP diameter, measurements were taken (20 repetitions) using a Nikon Eclipse 80i microscope with NIS Elements v. 3.22 software.

### 2.4. Biomass Production

To assess the mean MP dry weight of stock suspensions, 1 mL of fresh biomass was taken from storage (4 °C), placed in Petri dishes, and dried in an incubator (Nova Ética®, model 411d, Vargem Grande Paulista, São Paulo, Brazil) at 25 °C for 2 days. This assay was repeated 6 times. The dry weight was calculated in mg mL^−1^.

The MP reproductive capacity was then evaluated monthly (10 repetitions for each month) for one year by plating out 500 µL of MP biomass stock onto the potato dextrose agar culture medium in Petri dishes, spread uniformly with the aid of a Drigalski spatula, and incubated at 27 °C. The conidia produced were harvested with the aid of a spatula, and the plates were rinsed with 10 mL sterile 0.03% Tween 80 (*v*/*v*) to remove all remaining conidia from the plates. The conidial concentration was determined using a hemocytometer.

### 2.5. Bait Production

Baits were produced using powdered orange peel as a base. Oranges were peeled and the peel was dried in a forced air-drying chamber (Nova Ética® model 411d) at 28 °C for 48 h and then ground using an electric blender. A stainless-steel sieve with a 1 mm aperture was used to establish a homogenous powder. Baits were composed of powdered orange peel (90%), carboxymethylcellulose (5%), and soy oil (5%). All material was autoclaved for 20 min at 121 °C. After sterilization, 500 µL of fresh MPs in 10 mL sterile 0.03% Tween 80 or a 10 mL suspension of 1 × 10^7^ conidia mL^−1^ in 0.03% Tween 80 was added to each bait portion to be offered to the mini-colonies. After blending all components, bait pellets were produced with the aid of a sterile disposable syringe and then dried for 4 h at 27 °C in an air-drying chamber to remove all remaining water. Control baits were produced likewise, but without fungi.

### 2.6. Mini-Colony Bioassays

Baits were offered to mini-colonies to assess the effect of exposure of the fungus gardens to MPs in comparison to conidia and to verify the influence of viable and dead propagules on bait acceptance by worker ants. Five treatments were established, with five repetitions each: (1) viable MPs, (2) viable conidia, (3) dead MPs, (4) dead conidia, and (5) controls. In treatments 3 and 4, the MPs and conidia were autoclaved at 121 °C for 20 min prior to the bait production. The control treatment consisted of sterile distilled water + Tween 80 (0.03%). 

During the bioassay period, fresh *A. wilkesiana* leaves were offered daily for 7 days to mini-colonies, except on the 8th day, when 3 g of bait pellets were placed in the foraging area of each mini-colony, and during that time, no leaves were offered to the ants. Twenty-four hours later (day 9), all remaining bait was removed and quantified in order to establish the acceptance rate. After the baits had been offered, the bioassays continued for the next 15 days with fresh leaves offered every day. The foraging activity was recorded daily as the fresh leaf area (cm^2^) cut by workers, established from the difference between the initial leaf area and the remaining leaf area on the following day, which was estimated using a CI-202 portable leaf area meter (CID Bio-Science Inc., Camas, WA, USA).

The waste pile was removed daily from the plastic trays with the aid of a spatula to estimate the waste disposal rate. For the waste disposal analysis, bioassays were divided into 3 periods: from day 1 to day 7 (time 1), from day 8 to day 15 (time 2), and from day 16 to day 23 (time 3). The fungus garden weight was determined every 3 days. To do this, the plastic pots containing the fungus gardens were temporarily sealed to prevent workers from escaping and weighed with the aid of an analytical balance before returning the pots to the trays.

### 2.7. Statistical Analyses

The experimental design was completely randomized. Experimental units were mini-colonies that were randomly established from field colonies maintained under laboratory conditions. The variables analyzed were the (1) MP reproductive capacity, (2) bait acceptance rate, (3) foraging activity, (4) waste disposal, and (5) fungus garden weight. The treatments established were (1) viable MPs, (2) viable conidia, (3) dead MP, (4) dead conidia, and (5) control baits. For variable 1, ten repetitions (120 experimental samples) were performed and five repetitions of variables 2, 3, 4, and 5, with a total of 20 experimental samples each. A normality test (Shapiro–Wilk) was used to evaluate all variables.

To compare the waste disposal rate (g) between periods and between treatments, values for the mean waste disposal were subjected to a two-way ANOVA followed by Tukey’s post hoc test (α = 0.05). To compare the mean bait acceptance rate, fungus garden weight, and foraging activity, data were subjected to a one-way ANOVA followed by Tukey’s post hoc test (α = 0.05). The MP reproductive capacity was subjected to Kruskal–Wallis analysis, followed by Tukey’s post hoc test (α = 0.05), as it did not pass the normality test. All statistical analyses were carried out using Sigma-Plot (v. 12.5) software.

## 3. Results

### 3.1. MP Production in Liquid Culture

**Figure 1:**
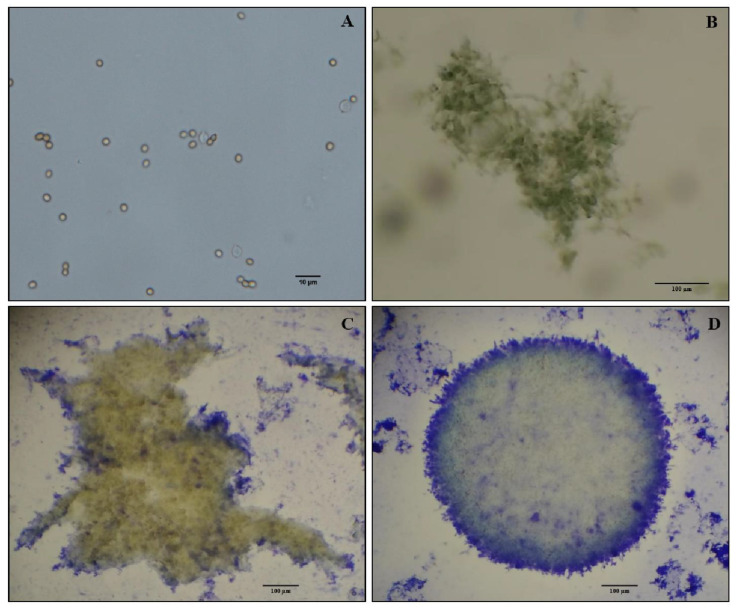
*Escovopsis weberi* MP development in liquid culture using an orbital shaker at 27 °C and 230 rpm: (**A**) conidial inoculation in liquid culture; (**B**) hyphae aggregating at 48 h; (**C**) early MP formation at 72 h; (**D**) complete MP formation at 96 h. (**A**): scale bar is 10 µm; (**B**–**D**): scale bar is 100 µm.

After four days, *E. weberi* conidia (Figure 1A) that had been inoculated in liquid medium produced MPs with variable sizes (57.47 to 685.86 µm in diameter), with a mean value of 280.82 µm (SEM ± 35.69). Hyphal development could be observed 48 h after conidial inoculation (Figure 1B,C). The liquid culture with chickpea flour, yeast extract, and dextrose induced the production of MPs with a compact structure (Figure 1D), and no melanization was observed at any time. Each milliliter of MP suspension had a mean dry weight of 0.411 mg (SEM ± 0.05). The MPs had a high capacity to produce conidia. Productivity assays showed that for each 500 µL of fresh MP biomass placed in Petri dishes and incubated for 5 days, 1 × 10^9^ conidia mL^−1^ (SEM ± 8 × 10^7^) was produced.

The viability of the MPs was evaluated monthly and statistical differences were observed over time (H_11_ = 64.995, *p* < 0.001) (Table 1). However, a statistical difference was only observed after six months. Conidia production remained stable during the first five months, with a range of 1.4 × 10^9^ to 1.1 × 10^9^ conidia mL^−1^ (Table 1). However, during months 10, 11, and 12 of the bioassay, the productivity dropped to 1.3 × 10^8^, 3.3 × 10^8^, and 4.6 × 10^8^ conidia mL^−1^, respectively.

### 3.2. Mini-Colony Bioassays

**Figure 2:**
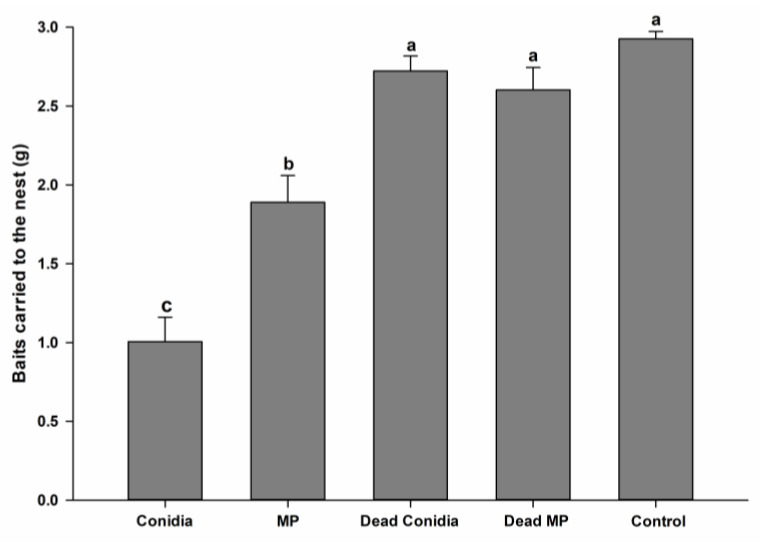
Mean weights of the baits transported by ant workers during a 24 h period. Different letters (a–c) indicated significant differences between treatments according to an ANOVA and Tukey’s test (α = 0.05).

The baits containing *E. weberi* offered to *A. subterraneus subterraneus* mini-colonies on the eighth day of the bioassay showed different acceptance patterns between the treatments. Baits containing viable conidia were the least accepted, with a mean of 1.006 g carried to the nest (F_4,20_ = 36.964, *p* < 0.001, normality test *p* = 0.708) (Figure 2). Viable MP baits were more acceptable to worker ants (1.890 g carried), and the weight of the baits carried to the nest was statistically different (*p* < 0.001) from that of viable conidia baits carried by the ants. Dead conidia (2.722 g), dead MP (2.602 g), and control (2.926 g) baits were highly acceptable and readily carried to the nest, with no statistical differences observed between these treatments (Figure 2).

### 3.3. Leaf Area Cut following Bait Exposure

**Figure 3:**
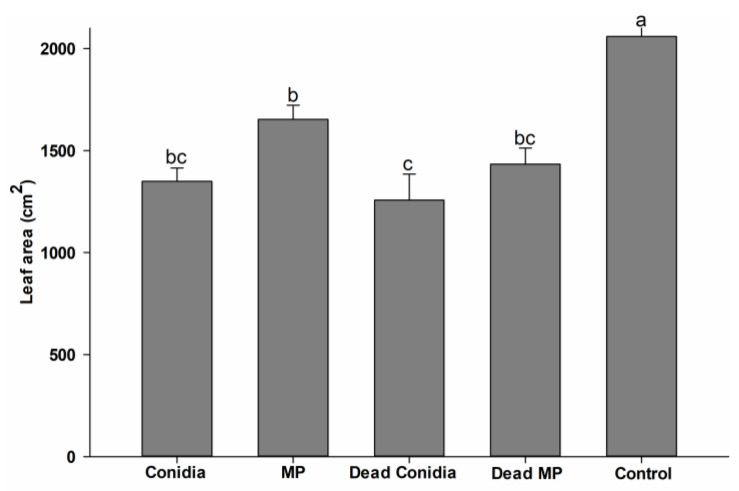
Cumulative daily foraging activity (leaf area cut (cm^2^)) performed by *A. subterraneus subterraneus* workers when offered *A. wilkesiana* during the bioassay period. Different letters (a–c) indicated significant differences between treatments according to Tukey’s test (α = 0.05).

Daily foraging activity (leaf area cut) showed slight differences between treatments that contained fungi (F_4,20_ = 14.622, *p* < 0.001, normality test *p* = 0.174) (Figure 3). The leaf areas cut following exposure to baits with viable MP were significantly different from those of mini-colonies exposed to dead conidia, but the viable conidia and dead MP treatments were not statistically different. Control treatments differed significantly from all other treatments (*p* < 0.001), with the highest foraging activity observed here (Figure 3). 

### 3.4. Waste Disposal

Before offering the baits (time 1), all treatments presented similar amounts of waste disposed of in the rubbish tip (F_4,60_ = 57.467, *p* < 0.001, normality test *p* = 0.141) (Table 2). After offering dead conidia, dead MP, and control baits (times 2 and 3), the waste disposal quantities remained similar to those prior to offering baits (time 1). However, for viable conidia and viable MP treatments, there were significant increases in the waste disposal rates during time 2 and time 3 (F_2,60_ = 17.302, *p* < 0.001; Table 2).

### 3.5. Fungal Garden Biomass

**Figure 4:**
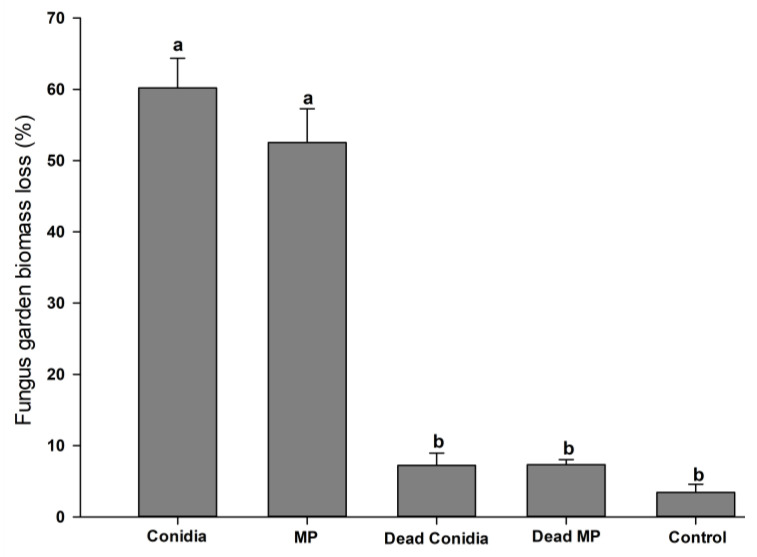
Fungal garden biomass loss (%) during the 23 days of the bioassay. Different letters (a,b) indicated significant differences between treatments according to Tukey’s test (α = 0.05).

Loss in fungal garden biomass could also be observed for colonies treated with viable conidia or viable MPs. From day one until the end of the bioassay, viable-conidia- and viable-MP-treated colonies lost 60.2% and 52.53% of their fungal garden biomass, respectively (F_4,20_ = 86.615, *p* < 0.001, normality test *p* = 0.479) (Figure 4). Conversely, loss of fungal garden biomass when colonies were exposed to dead conidia, dead MP, or control treatments was very low and did not present statistical differences when comparing these three groups. The biomass losses were 7.23%, 7.33%, and 3.44% (*p* > 0.005) in these three treatments, respectively (Figure 4). 

## 4. Discussion

Here, it is reported for the first time that *E. weberi*, when cultured in a liquid medium, can produce hyphal aggregations regarded to be mycelial pellets (MPs). Under laboratory conditions, it is possible to induce certain species of fungi to produce these types of propagules [53,54,55]. The medium used here was originally developed to stimulate the production of blastospores by *Metarhizium anisopliae* [52]. However, in the current study, the protocol was adapted to induce the production of MPs by *E. weberi.*

*Escovopsis weberi* grown in a liquid medium produced MPs of variable sizes, similar to those seen in previous studies of entomopathogenic fungi [45,47,56]. *Escovopsis weberi* MP presented faster germination than conidia when plated onto a solid culture medium (unpublished data). It is normal for MPs to germinate rapidly in favorable conditions due to the high numbers of mitochondria and high levels of polysaccharides and lipids in the cytoplasm of the hyphal cells [57]. As observed in this study, when using a PDA culture medium, the MPs germinated and formed conidiphores, which subsequently resulted in high concentrations of conidia. The MPs maintained a high level of conidial productivity over a 12-month storage period, with only the last three months presenting statistical differences from the previous months, but the results still demonstrated high levels of conidial production. This result is important when considering the shelf life and stability of this type of propagule, increasing its potential as a biological control agent.

Sclerotia found in soils and plant material generally present as dark brown or black due to the presence of melanin [44], different from mycelial pellets produced in liquid culture under constant agitation, which are mostly light brown [49,58]. Likewise, the *E. weberi* MPs obtained in this study were light brown, with no melanization observed during any of the developmental stages.

In *A. subterraneus subterraneus* mini-colonies, bait acceptance rates of worker ants offered viable and dead *E. weberi* conidia or MPs were assessed. We observed that the ants could distinguish between the type of propagule (conidia or MP) due to the significant differences in acceptance rates. Workers carried a lower percentage of conidial baits to the fungal garden in comparison to the others treatments. Some studies showed that *Escovopsis* can enter the colony as conidia present in the environment attached to arthropod bodies [41,59,60]. Augustin et al. [60] showed that *Escovopsis* conidia enter the ant colonies through accidental contact of workers with spores when spores are present in foraging areas around the nest. They also observed that workers were not able to immediately recognize such small quantities of conidia on their bodies and, therefore, did not carry out self-grooming. Thus, the high conidial concentration employed in the present study may explain its detection by worker ants, even though the orange-based baits were highly attractive, which may disguise the pathogen [61]. These results were similar to those seen by Augustin et al. [60].

Fungal hyphae and conidia have certain similarities and certain differences in chemical composition. While both propagules have polysaccharides, such as β-1,3 glucan and chitin, in their cell walls, conidia are covered by a protective layer containing I-hydrophobin RodA and DHN-melanin [62]. These two compounds are critical in providing hydrophobicity and tolerance to environmental conditions [63,64]. It is possible that the difference in acceptance patterns observed in this study between conidia and MPs was influenced by differences in the cell wall composition of these propagules, i.e., ants may recognize the compounds in conidia as a greater threat to the colony. More studies on the differences between conidia, hyphae, MP cell walls, and their recognition by ants are necessary.

Leaf-cutting ants can forage a wide variety of plant material at high speed. They carry the plant material into their nest and incorporate it into the fungal garden. This capacity to cut large quantities of fresh leaves makes leaf-cutting ants one of the most important herbivores in the Neotropics, causing damage and economic losses in agricultural systems [1,3,11,12,13,64]. Leaf-cutting ants are very selective regarding the plant material they carry to the nest and have the capacity to recognize organisms or substances that are harmful either to individuals or to their symbiont fungus [3,65]. This factor is one of the main challenges when using baits to control these insects, as the ants may refuse the baits containing biological control agents. Thus, it is important to develop strategies that can effectively deliver these agents to the ants.

Important differences were observed in the acceptance rates of baits incorporating dead or viable propagules. Almost 100% of the bait containing both types of dead propagules was carried to the nest by workers. In contrast, baits with viable propagules were significantly less likely to be accepted. Despite the differences in the cell walls of conidia and hyphae mentioned above, it is possible that viable fungi may produce volatile organic compounds that are detected by worker ants. Such volatiles may be absent in dead fungal propagules. Fungi naturally produce volatile compounds for different purposes, such as promoting conidiation, recognition, or behavioral changes [66,67,68]. Kandasamy et al. [66] observed that *Ips typographus*, which is a bark beetle that attacks pine trees in coniferous forests, can distinguish its symbiotic fungus from other fungi through volatile organic compounds emitted by the former. Thus, the beetle is correctly attracted to food sources colonized by the symbiont. Future studies concerning insect–fungus recognition in leaf-cutting ants and their associated fungus may clarify the observations found in this study.

Although fungal viability had a marked influence on bait acceptance, it did not have the same effect on the foraging activity. In all treatments containing fungi, viable or not, the *A. wilkesiana* accumulated leaf area (cm^2^) offered daily to mini-colonies that was cut by workers was not statistically different. However, in the control treatments, the total leaf area cut was higher than in all fungal treatments. This observation indicated that the presence of *Escovopsis*, viable or not, modified the foraging behavior of the ants. This behavior strategy may be an attempt to prevent a possible *Escovopsis* reintroduction into the colony [65].

The presence of *Escovopsis* baits in the nest influenced the amount of waste disposed of by the ants. After worker ants tended the fungal garden into which foragers had carried baits containing both types of viable propagules, the amount of waste disposed of by the ants increased notably in comparison to assays with both types of dead propagules and control treatments. This may be associated with the presence of *Escovopsis* in the colonies. Behaviors such as fungus grooming and weeding, which are done to prevent colony collapse, are commonly observed in colonies infected by *Escovopsis* [38]. Once the colony has been contaminated with *Escovopsis*, the ants attempt to free the fungus garden from infection by gathering *Escovopsis* spores and sterilizing them in their infrabuccal pockets [37,69,70,71]. Workers also detach and dispose of portions of the fungal garden contaminated with the parasite. In both cases, workers deposit the alien fungus or the contaminated garden fragments in the rubbish tips [38]. 

Despite the effort of fungus grooming and weeding, *Escovopsis* can still dominate the colony. It was shown that only *Escovopsis* conidia are inhibited by metapleural glands [35]. However, after conidial germination, *Escovopsis* hyphae are no longer affected by the compounds produced by this gland. In addition, once *Escovopsis* hyphae begin to develop and grow inside the colony, *Escovopsis* produces substances such as melinacidin IV and shearinine D, both of which are lethal to *Pseudonocardia* [72]. The non-susceptibility of *Escovopsis* hyphae to metapleural gland secretions and the inhibition of *Pseudonocardia* indicate that this parasite employs these strategies to successfully colonize its host. As a means to control *Escovopsis* infection, worker ants increase weeding and fungus grooming activities, which, in turn, increases the waste disposal rate and decreases the fungal garden biomass, as observed in the current study. There was a clear influence of these two factors during the bioassays using viable conidia and MPs. This pattern of increased waste disposal and decreased fungus garden biomass was not recorded for dead propagules or control treatments.

Previously, there were no reports of MP production by myco-parasitic fungi, such as *Escovopsis*. *Escovopsis weberi* is a specialist parasite of *Leucoagaricus gongylophorus* and is a threat to leaf-cutting ant colonies [59]. The use of entomopathogenic, phytopathogenic, and nematophagous fungal MPs for biological control was studied and shown as a potential alternative to chemical control [45,46,47,55,58,73,74]. The application of *Escovopsis* MPs could also have the potential for use in biological pest control programs due to its capacity to affect colony integrity and remain viable for long periods.

## 5. Conclusions

This is the first report of mycelial pellet production by *Escovopsis weberi*. Here, we observed that fresh and stored MPs were able to rapidly germinate and produce conidia. Baits containing MPs were more attractive to leaf-cutting ants than baits incorporating conidia, indicating the potential of MPs for use in biocontrol strategies with a lower risk of rejection. MPs had a negative effect on the fungus garden, resulting in a deterioration in colony health as measured by the sharp decline in biomass.

## Figures and Tables

**Table 1:** Monthly mycelial pellet reproductive capacity (conidia mL^−1^) during a 12-month storage period.

Evaluation Time (Months)	Median (×10^8^)
1	11.68 a
2	12.40 a
3	11.72 a
4	14.54 a
5	12.64 a
6	9.67 abc
7	10.23 ab
8	9.44 abc
9	10.05 abc
10	1.3 d
11	3.35 cd
12	4.68 bcd

Medians followed by different letters (a–d) were statistically different to each other according to the Kruskal–Wallis test, followed by Tukey’s test (α = 0.05).

**Table 2:** Waste weight in grams (mean ± SEM) dumped during 23 days of the bioassay. From day 1 to day 7 (time 1), day 8 to day 15 (time 2), day 16 to day 23 (time 3).

Treatments	Times		
	1	2	3
Conidia	0.20 ± 0.05 Ba	0.58 ± 0.05 Aa	0.51 ± 0.03 Aa
MP	0.21 ± 0.05 Ba	0.56 ± 0.05 Aa	0.47 ± 0.02 Aa
Dead Conidia	0.15 ± 0.16 Aa	0.17 ± 0.02 Ab	0.16 ± 0.01 Ab
Dead MP	0.21 ± 0.02 Aa	0.20 ± 0.01 Ab	0.20 ± 0.02 Ab
Control	0.16 ± 0.02 Aa	0.19 ± 0.02 Ab	0.17 ± 0.02 Ab

Uppercase letters (A,B): comparisons between periods; lowercase letters (a,b): comparisons among treatments. Means followed by different letters indicated statistical differences according to Tukey’s test (α = 0.05).

## Data Availability

The sequence data is available at NCBI under accession numbers OQ345555 and OQ345556.
